# Establishment of a Novel In Vitro Model of Endometriosis with Oncogenic *KRAS* and *PIK3CA* Mutations for Understanding the Underlying Biology and Molecular Pathogenesis

**DOI:** 10.3390/cancers13133174

**Published:** 2021-06-25

**Authors:** Mohammad Mahmud Hossain, Kentaro Nakayama, Kamrunnahar Shanta, Sultana Razia, Masako Ishikawa, Tomoka Ishibashi, Hitomi Yamashita, Seiya Sato, Kouji Iida, Kosuke Kanno, Noriyoshi Ishikawa, Tohru Kiyono, Satoru Kyo

**Affiliations:** 1 Department of Obstetrics and Gynecology, Shimane University Faculty of Medicine, Izumo 693-8501, Japan; likhon.vet@gmail.com (M.M.H.); kamrunnahar.vet@gmail.com (K.S.); raeedahmed@yahoo.com (S.R.); m-ishi@med.shimane-u.ac.jp (M.I.); tomoka@med.shimane-u.ac.jp (T.I.); meme1103@med.shimane-u.ac.jp (H.Y.); sseiya@med.shimane-u.ac.jp (S.S.); iida@med.shimane-u.ac.jp (K.I.); kanno39@med.shimane-u.ac.jp (K.K.); satoruky@med.shimane-u.ac.jp (S.K.); 2 Department of Organ Pathology, Shimane University Faculty of Medicine, Izumo 693-8501, Japan; kanatomo@med.shimane-u.ac.jp; 3 Project for Prevention of HPV-Related Cancer, Exploratory Oncology Research and Clinical Trial Center (EPOC), National Cancer Center, Kashiwa 277-8577, Japan

**Keywords:** ovarian endometriosis, epithelial cell line, PIK3CA, KRAS, invasion, migration, cell proliferation, microarray analysis, LOX, PTX3

## Abstract

**Simple Summary:**

Endometriosis is a common gynecological condition that causes pelvic pain and infertility. Despite having normal histological features, several cells bear cancer-associated somatic mutations that result in local tissue invasion but rarely metastasize. Several cancer-associated genes, such as *KRAS* and *PIK3CA*, are frequently mutated in the endometriotic epithelium. However, the functional behavior and molecular pathogenesis of this disorder remain unclear. In this study, we developed an immortalized endometriotic epithelial cell line with mutations in *KRAS* and *PIK3CA*, which are genes associated with aggressive behaviors, such as increased cell migration, invasion, and proliferation. Through microarray analysis, the *KRAS*- and *PIK3CA*-specific gene signatures were identified; *LOX* and *PTX3* were found to be responsible for this metastatic behavior. Knockdown of these two genes by siRNA markedly reduced the metastatic ability of the cells. Our findings suggest that inhibition of *LOX* and *PTX3* may be an alternative therapeutic strategy to reduce the incidence of endometriosis.

**Abstract:**

Endometriosis-harboring cancer-associated somatic mutations of *PIK3CA* and *KRAS* provides new opportunities for studying the multistep processes responsible for the functional and molecular changes in this disease. We aimed to establish a novel in vitro endometriosis model to clarify the functional behavior and molecular pathogenesis of this disorder. Immortalized HMOsisEC10 human ovarian endometriotic epithelial cell line was used in which *KRAS* and *PIK3CA* mutations were introduced. Migration, invasion, proliferation, and microarray analyses were performed using *KRAS* and *PIK3CA* mutant cell lines. In vitro assays showed that migration, invasion, and proliferation were significantly increased in *KRAS* and *PIK3CA* mutant cell lines, indicating that these mutations played causative roles in the aggressive behavior of endometriosis. Microarray analysis identified a cluster of gene signatures; among them, two significantly upregulated cancer-related genes, lysyl oxidase (*LOX*) and pentraxin3 (*PTX3*), were associated with cell proliferation, invasion, and migration capabilities. Furthermore, siRNA knockdown of the two genes markedly reduced the metastatic ability of the cells. These results suggest that endometriosis with *KRAS* or *PIK3CA* mutations can significantly enhance cell migration, invasion, and proliferation by upregulating *LOX* and *PTX3*. We propose that *LOX* and *PTX3* silencing using small molecules could be an alternative therapeutic regimen for severe endometriosis.

## 1. Introduction

Endometriosis is a benign inflammatory disease, characterized with the recurrent estrogen-dependent ectopic growth of the endometriotic epithelium and stroma outside the uterus. It affects approximately 10–15% of the women of reproductive age [1,2], causing pelvic pain and infertility [2,3]. Despite its low mortality rate, endometriosis has a significant impact on the quality of life and work productivity and is a substantial socioeconomic burden on the healthcare system [4,5]. Endometriosis has been clinically recognized for more than a century, but its pathogenesis and mechanism of dissemination remain unclear. The current prevailing hypothesis is that endometriosis results from retrograde menstruation, causing the endometriotic cells (composed of epithelial glands and stromal cells) to adhere to the pelvic peritoneum, ovaries, and rectovaginal septum, with subsequent implantation and proliferation until they form endometriotic lesions [6,7]. Anatomically, endometriosis has three major manifestations in the pelvic region: superficial, ovarian, and deep infiltrating [8,9]. Although it was previously considered a benign condition with normal-acting histological features, recent studies have established that endometriosis can recapitulate some cancer-like features, such as cell proliferation, migration, and invasion. Furthermore, one study showed that recurrently mutated cancer driver genes, such as *KRAS*, *PIK3CA*, *PPP2R1A*, and *ARID1A*, are also present in endometriosis without cancer association [3]. Consequently, ovarian endometriosis may be considered a precursor of endometrioid ovarian carcinoma and clear cell carcinoma [10,11,12,13]. In addition, some studies have suggested that mucinous metaplasia in ovarian endometriosis could be closely linked to the development of borderline mucinous ovarian carcinoma with *KRAS* mutation, which may eventually progress to invasive carcinoma [14,15]. Recently, some studies have revealed that ovarian endometriosis, deep-infiltrating endometriosis, uterine adenomyosis, and normal uterine endometrium harbored cancer-associated somatic mutations [3,16,17,18,19,20,21]. Additionally, *PIK3CA* or *KRAS* mutated clones arising in the histologically normal uterine endometrium have been proposed as the cellular origins of endometriosis [3]. The transformation of a normal cell into a cancer cell is caused by the acquisition of driver mutations and epigenetic modifications, accompanied by changes in the cellular morphology and tissue architecture [22]. However, no behavioral and functional analysis of the aforementioned genes in endometriosis has been performed.

In the current study, we aimed to comprehensively analyze the roles of *KRAS* and *PIK3CA* oncogenes in endometriosis. To this end, we established an immortalized cell line model from ovarian endometriotic epithelial cells to perform in vitro cellular functional analyses (migration, invasion, and proliferation assays). In addition, we performed microarray analyses of immortalized endometriotic epithelial cell lines (*KRAS* and *PIK3CA* mutant) to identify migration and invasion-related gene signatures. To the best of our knowledge, this is the first study to perform a functional analysis of *KRAS* and *PIK3CA* mutant endometriotic epithelial cells.

## 2. Results

### 2.1. Development of Immortalized Epithelial Cell Lines from Ovarian Endometriotic Tissue

Surface epithelial tissue of ovarian endometriosis was collected from a 48-year-old patient undergoing laparoscopic ovarian cystectomy as treatment for ovarian endometriosis. To determine whether mixing of malignant and our collected sample had occurred, we conducted further pathological examination and found no malignant lesions. In a previous study, it was found that the triple expression of an active *CDK4* mutant (CDK4^R24G^), *cyclin D1*, and *hTERT* were sufficient to immortalize epithelial cells [23]. Based on this, we also introduced these factors into our isolated endometriotic cells and observed their morphology. We noted the absence of morphological changes in the immortalized ovarian endometriotic epithelial cells, along with faster growth and overcoming of premature senescence. We named these cell lines HMOsisEC10 (Wild type), HMOsisEC10 *KRAS* mutant and HMOsisEC10 *PIK3CA* mutant cell lines (Figure S1).

### 2.2. Western Blot Analysis of pan-Cytokeratin, pan-AKT, pan-MAPK, p-MAPK, and p-AKT Expression in Immortalized Cells

To confirm the epithelial origin of the immortal endometriotic cells, Western blotting and immunocytochemistry using pan-cytokeratin were performed. The results showed that the epithelial morphology was highly detectable in the HMOsisEC10 immortal cell line (Figure S2). We subsequently focused on the status of the *KRAS* and *PIK3CA* mutations. Western blot assays showed that the RAS/ERK signaling pathway was strongly activated in the HMOsisEC10 *KRAS* mutant cells, whereas the PI3K/AKT signaling pathway was activated in the HMOsisEC10 *PIK3CA* mutant cells (Table S1 and Figure S3).

### 2.3. Short Tandem Repeat (STR) Analysis

Maintaining genetic stability in continuously cultured cells is critical for life science research. We performed STR analysis to evaluate the integrity (genetic origin and cross-contamination) of the immortalized HMOsisEC10 cell line. Specifically, we tested DNA samples from HMOsisEC10 early and late passages 2, 13, and 22. Eight STR loci (TPOX, CSF1P0, VWA, FGA, D3S1359, D18S51, D8S315, and SE33) per sample were amplified. We observed that cells from all three passages had identical STR profiling patterns in the eight loci (Figure S4). This result suggests that cross-contamination was absent across these cell lines.

### 2.4. Migration, Invasion, Proliferation, and Anchorage-Independent Assays of Mutant Cells

**Figure 1:**
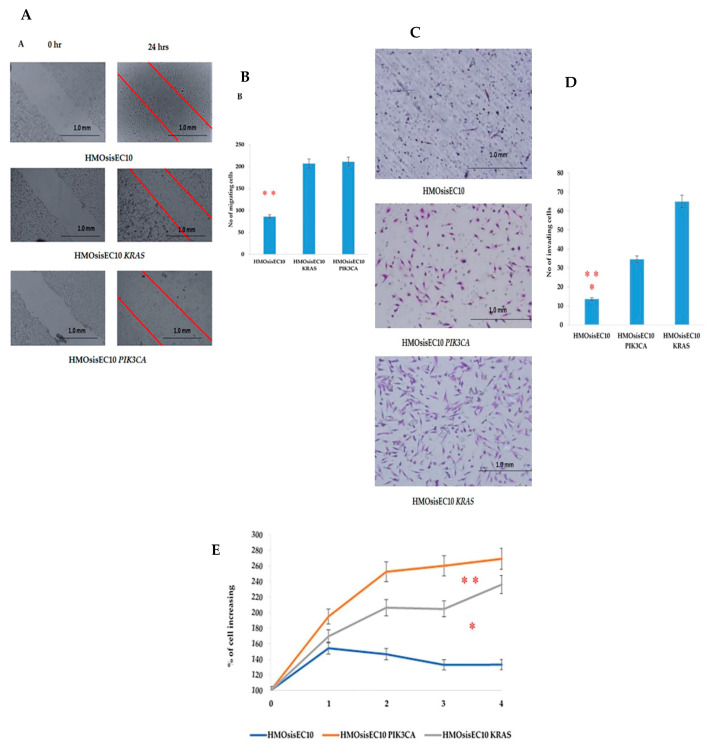
HMOsisEC10 *KRAS* and HMOsisEC10 *PIK3CA* cells exhibit increased migration, invasion, and proliferation relative to HMOsisEC10 cells. Cell migration assay (**A**,**B**). HMOsisEC10 *KRAS* and HMOsisEC10 *PIK3CA* cell lines show increased cell migration in the wound healing assay. Photographs captured immediately after the scratch (0 h, upper left panel) and 24 h post-scratch (upper right panel). The number of migrated cells is significantly higher in the HMOsisEC10 *KRAS* and HMOsisEC10 *PIK3CA* mutant cell lines than in the HMOsisEC10 cell line (** *p* < 0.05). Matrigel invasion assay (**C**,**D**). The HMOsisEC10 *KRAS* and HMOsisEC10 *PIK3CA* mutant cell lines demonstrate a significantly higher invasion capacity than the control HMOsisEC10 cell line (** *p* < 0.01 and * *p* < 0.05, respectively). Cell proliferation assay (**E**). The proliferation capability of the mutant HMOsisEC10 *KRAS* and HMOsisEC10 *PIK3CA* cell lines are significantly higher than that of the HMOsisEC10 cell line (* *p* < 0.05 and ** *p* < 0.01, respectively). The error bars indicate standard deviation.

To check the cellular functional behavior, we conducted an in vitro migration assay by using the immortalized cell lines we established. The results showed that the HMOsisEC10 *KRAS* and HMOsisEC10 *PIK3CA* mutant cell lines had significantly higher migration ability than the HMOsisEC10 cell line (Figure 1A,B). We next examined the invasion behavior of the cells using the Matrigel invasion assay. In this assay, cells are required to invade the extracellular matrix barrier. FBS (20%) was used as a chemoattractant, and cell invasion was assessed after 24 h. The HMOsisEC10 *KRAS* and HMOsisEC10 *PIK3CA* mutant cell lines demonstrated significantly increased invasion ability compared to the HMOsisEC10 cell line (Figure 1C,D). The cellular proliferation assay revealed a similar result, where HMOsisEC10 *KRAS* and HMOsisEC10 *PIK3CA* mutant cells exhibited significantly increased proliferation compared to HMOsisEC10 (Figure 1E). To evaluate the transformation phenotype, an anchorage-independent assay was performed, which showed no colonies in either the HMOsisEC10 *KRAS* or HMOsisEC10 *PIK3CA* mutant cell lines. SKOV3 was simultaneously examined as a positive control where several distinct colonies were identified (Figure S5).

### 2.5. Constitutive Effect of Tumorigenicity in HMOsisEC10 KRAS and HMOsisEC10 PIK3CA Mutant Cells

Having established the phenotypic changes in the HMOsisEC10 *KRAS* and HMOsisEC10 *PIK3CA* mutant epithelial cell lines, we next examined whether these cells showed altered tumor growth in nude mice. Subcutaneous injections of HMOsisEC10 *KRAS* and HMOsisEC10 *PIK3CA* mutant cells in nude mice did not produce any forms of tumor within two months of monitoring. Besides, SKOV3 cells were inoculated as positive control and exhibit tumor (Figure S6).

### 2.6. Lack of Other Genetic Mutations in Immortalized Epithelial Cells

We next performed the whole-exome sequencing to identify whether the immortal HMOsisEC10 cell line had acquired any tumor-specific somatic mutations. We evaluated the mutation burden of 728 cancer-related genes and the genomic DNA analysis results were compared with the result from normal endometriotic tissue. The result revels that there were no cancer specific alterations in somatic genes, including single-nucleotide variations, insertions, or deletions. The results also found no copy number alterations in oncogenes, or deficiencies in mismatch repair genes, homologous recombination genes, and tumor suppressor genes (Table S2 and Figure S7).

### 2.7. Identification and Analysis of Genes Involved in Cell Migration, Invasion, and Proliferation

**Figure 2:**
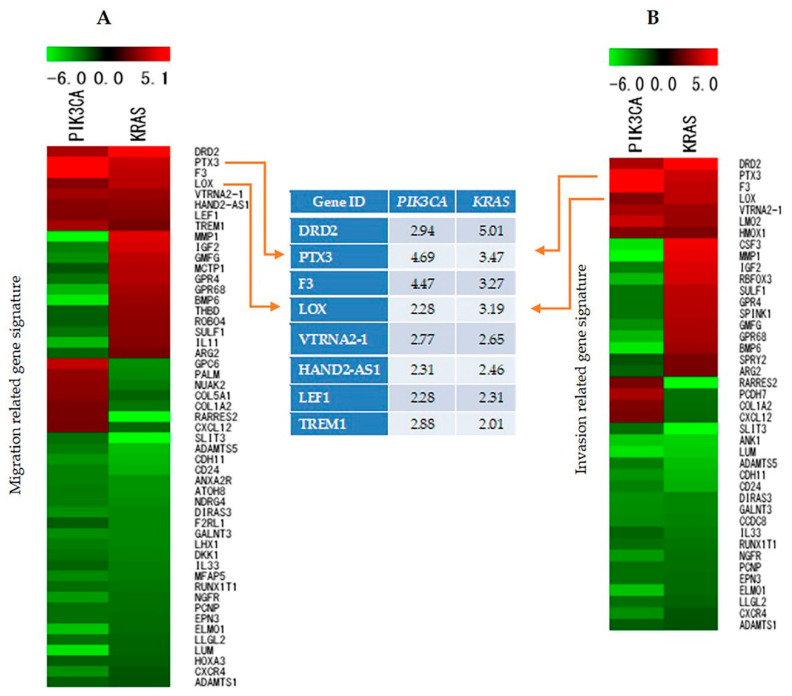
Gene expression analysis. Heat map representing expression pattern for migration (**A**) and invasion (**B**)-related gene signatures in the HMOsisEC10 *KRAS* and HMOsisEC10 *PIK3CA* mutant cell lines. Red color shows upregulated genes, and green color shows downregulated genes. Arrowhead indicates the genes selected for further analysis.

We performed microarray analysis with the aim of identifying the genes that are responsible for migration, invasion, and proliferation in HMOsisEC10 *KRAS* and HMOsisEC10 *PIK3CA* mutant cell lines. Microarray analysis revealed 5959 genes that displayed changes in the pattern of gene expression, such as increased or decreased expression in the three different cell lines. Among them, eight genes were found to be more than two-fold over-expressed, and 24 genes were under-expressed **in** both the HMOsisEC10 *KRAS* and HMOsisEC10 *PIK3CA* cell lines compared to the HMOsisEC10 cells in genes associated with migration (Figure 2A). With regard to invasion, heat map analysis revealed that 7 genes were more than two-fold over-expressed, whereas 18 genes were under-expressed in *KRAS* and *PIK3CA* mutant cells (Figure 2B). From this gene list, gene expression changes associated with migration and invasion potential were identified by conducting a comprehensive search using the PubMed database. Owing to their high migration and invasion ability, the *LOX* and *PTX3* genes were selected for further analysis from the top upregulated genes. Microarray analysis was also helpful to identify the pathways regulated in endometriosis in the HMOsisEC10 *KRAS* and HMOsisEC10 *PIK3CA* mutant cells.

### 2.8. Validation of Microarray Data by Reverse Transcriptase Polymerase Chain Reaction (RT-PCR)

To confirm the differential expression of the genes identified by cDNA microarray analysis of mRNA samples isolated from the *KRAS* and *PIK3CA* mutant cell lines, we analyzed two upregulated genes using RT-PCR. *GAPDH* expression was used as a control for the input cDNA. The RT-PCR results were consistent with those obtained from the microarray analysis, indicating that the microarray results provided an accurate report of the transcript levels (Figure S8).

### 2.9. Signaling Pathways Targeting KRAS and PIK3CA Mutations in Endometriosis Due to the Upregulation of Lysyl Oxidase (LOX) and Pentraxin3 (PTX3)

**Figure 3:**
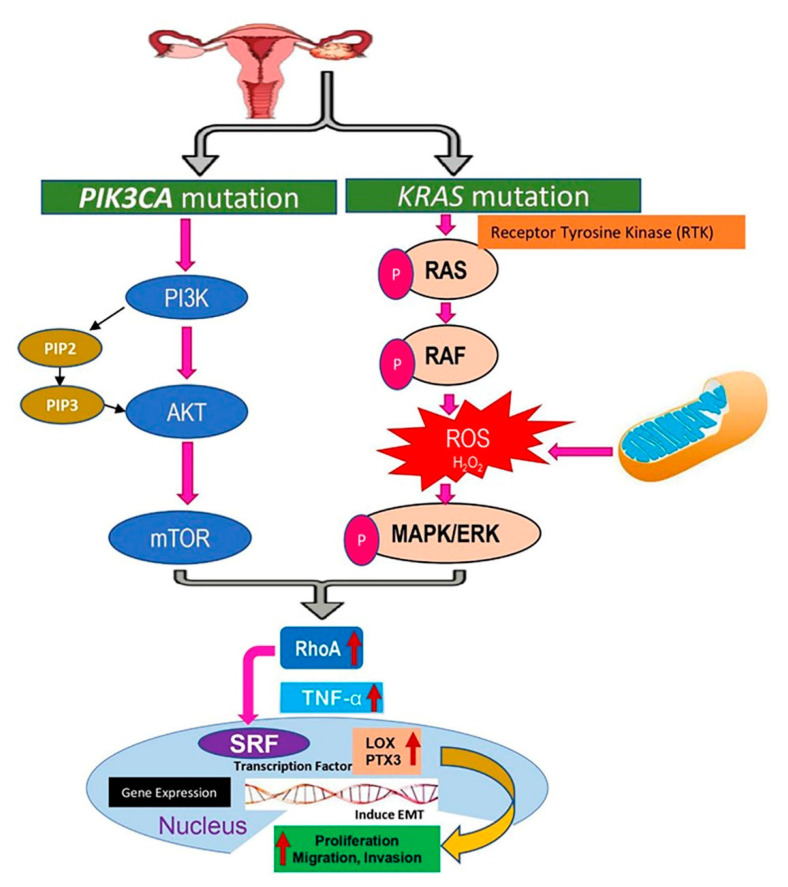
Targeting *KRAS* and *PIK3CA* mutations in endometriosis; *KRAS* mutation activates RAS, RAF, and MAPK/ERK with the aid of receptor tyrosine kinases through phosphorylation. PI3K is activated by the *PIK3CA* mutation, which results in phosphorylation of PIP2 to PIP3 and activates AKT and mTOR. MAPK/ERK and mTOR have common effects on RhoA activity, which regulates the serum response elements (SRF) with the help of TNF-α. SRF is a transcription factor that has downstream effects on *LOX* and *PTX3* leading to cell proliferation, migration, and invasion.

In order to recognize the pathways (Figure 3) that were regulated in endometriosis, a global microarray analysis was undertaken. Through this analysis, we recognized a cluster of genes and the associated pathways that automatically promoted cell proliferation, invasion, and migration in endometriotic epithelial cells by regulating mutant *KRAS* and *PIK3CA* expression. These genes included *LOX* and *PTX3*. Apart from these two genes, several principle regulators were present in this system, such as transcription factors, cell adhesion molecules, and microRNAs that may interact and stimulate the expression of these and several other genes. A previous report suggested that *KRAS* and *PIK3CA* mutations have a synergistic effect in activating the PI3K-AKT-mTOR pathway [24], and *KRAS* mutations activate the RAS and RAF proteins, which have the capability to regulate several signaling cascades important for cell proliferation, migration, and invasion [25], including the MAPK/ERK pathway. Notably, the PI3K-AKT-mTOR pathway and RAS/RAF/MAPK/ERK pathway have similar up regulation effects on RhoA activity (a Rho GTPase that acts as oncogene) [26]. The microarray data obtained in this study revealed upregulation of RhoA, TNF-α, and serum response factor (SRF) expression in *KRAS* and *PIK3CA* mutant cell lines (Table S3). The overexpressed RhoA stimulates cell cycle progression as well as enhances cell migration through activation of TNF-α [27,28]. The transcriptional factor SRF is activated by the influence of Rho subfamily [29], and upregulated *LOX* and *PTX3* might have positive effects on cell proliferation, migration, and invasion potential.

### 2.10. LOX and PTX3 mRNA Expression Levels Are Upregulated in Immortalized Endometriotic Mutant Epithelial Cells

**Figure 4:**
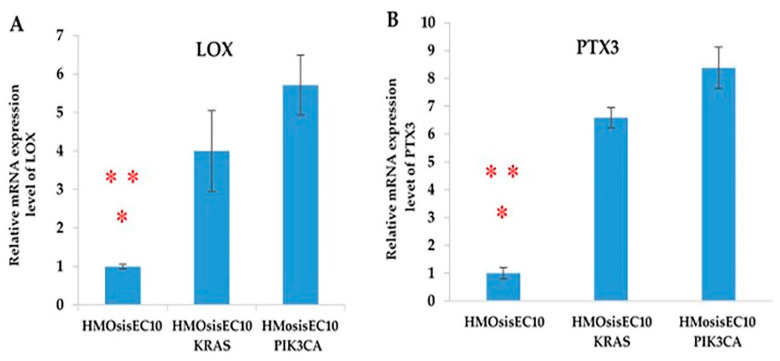
mRNA expression levels of LOX (**A**) and PTX3 (**B**) are evaluated in mutant cell lines (HMOsisEC10 *KRAS* and HMOsisEC10 *PIK3CA)*. RT-PCR results revealed that both mutant cell lines showed a significantly higher expression of *LOX* and *PTX3* compared to the HMOsisEC10 cell line (* *p* < 0.05 and ** *p* < 0.01). Error bars indicate standard deviation.

First, we determined the mRNA levels of *LOX* and *PTX3* in immortalized endometriotic epithelial cells using RT-PCR. Our results revealed the mRNA levels of *LOX* and *PTX3* in cells expressed as relative units after normalization with the housekeeping gene *GAPDH*. *LOX* and *PTX3* were highly expressed in the mutant cell lines (HMOsisEC10 *KRAS* and HMOsisEC10 *PIK3CA*) compared to the wild-type HMOsisEC10 cell line (Figure 4).

### 2.11. Constitutive Knockdown of LOX and PTX3 Leads to Decreased Migration, Invasion, and Proliferation in KRAS and PIK3CA Mutant Cell Lines

**Figure 5:**
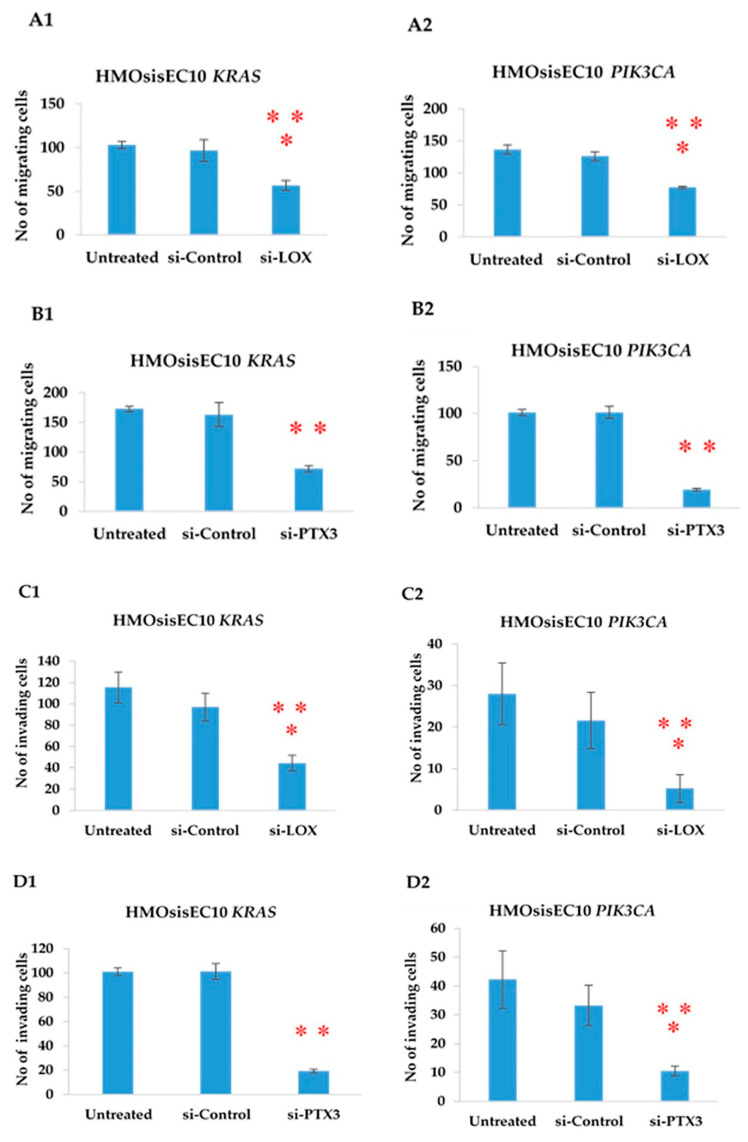

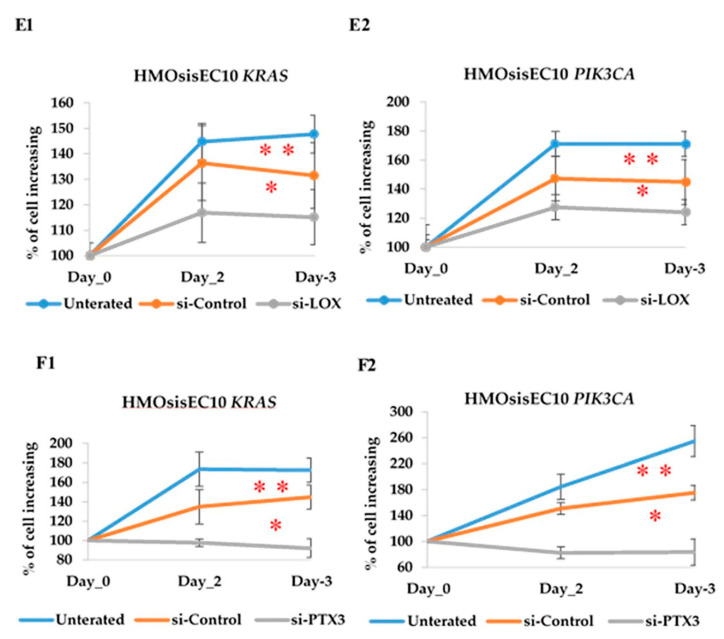
Cell migration assay. Migration abilities of mutant cell lines (HMOsisEC10 *KRAS* and HMOsisEC10 *PIK3CA)* are measured using a scratch wound healing assay after knockdown with *LOX* (**A1**,**A2**) and *PTX3* siRNA (**B1**,**B2**). The numbers of migrated cells were significantly lower in siRNA treated cells compared to the control siRNA and untreated cells. Matrigel Invasion assay. siRNA knockdown of *LOX* (**C1**,**C2**) and *PTX3* (**D1**,**D2)** showed a significantly lower invasion capacity than control siRNA and untreated cells. Cell proliferation assay. Treatment with *LOX* (**E1**,**E2**) and *PTX3* siRNA (**F1**,**F2**) significantly reduced cell proliferation ability relative to the control siRNA and untreated cells. ** *p* < 0.01 and * *p* < 0.05. The error bars indicate standard deviation.

To confirm that the overexpression of *LOX* and *PTX3* led to an increase in the migration, invasion, and proliferation ability in the *KRAS* and *PIK3CA* mutant cell lines, we applied a complementary approach using a gene knockdown system. HMOsisEC10 *KRAS* and HMOsisEC10 *PIK3CA* cells were transfected with siRNA (Santa Cruz Biotechnology) against *LOX* and *PTX3*, and the effect on cell migration was measured by using a wound healing assay after 48 h. Our data showed that siRNA knockdown of both *LOX* and *PTX3* significantly inhibited cell migration (Figure 5A1,2,B1,B2). We also examined whether the silencing of these genes could affect cellular invasion. Both *LOX* and *PTX3* significantly inhibited mutant cell invasion (Figure 5C1,C2,D1,D2). Similarly, we conducted a cell proliferation assay after silencing of *LOX* and *PTX3* for 48 h and found that cell proliferation was significantly reduced (Figure 5E1,E2,F1,F2). Other *LOX* and *PTX3* siRNAs (Ambion Life Technologies) were also tested using migration, invasion, and proliferation assays (Figure S9); siRNA sequences are listed in Table S4.

## 3. Discussion

Despite the high prevalence of endometriosis and its outcome on women’s health, very little is known about the biological processes that reinforce it, although more than an era has passed since this disease was first described [3,5]. Anglesio et al. reported that 26% of patients with deep-infiltrating endometriotic epithelial cells showed somatic cancer driver mutations, such as *KRAS*, *PIK3CA*, *PPP2RIA*, and *ARID1A* [3]. Moreover, ovarian endometriotic cysts and uterine endometrium also harbored cancer-associated somatic mutations [16,17]. Furthermore, *KRAS* mutations in different tissue areas have been reported, suggesting the presence of clonal heterogeneity and that a gradual process of changes in endometriosis is possible [3]. Among the targeted genes, *KRAS* and *PIK3CA* are most frequently mutated in the normal epithelial cells of the uterine endometrium and in endometriosis, with a higher mutant allele frequency (MAF). This increasing MAF in cancer-associated genes in the endometriotic epithelium recommend cancer-associated mutation already harboring in retrograde flow of endometrial cells before implantation/seeding, or they may independently arise in the implanted endometriotic epithelial cells thereby inducing the development of endometriosis [3,16,17,18,19,20,21]. Accordingly, our study aimed to investigate the functional behavior of these *KRAS* and *PIK3CA* cancer-driver genes along with identifying the somatic mutations associated with the aggressive benign tumor in the developed immortalized endometriotic epithelial cell line, HMOsisEC10. In addition, using microarray analysis, we also identified the overexpression of *LOX* and *PTX3* in *KARS* and *PIK3CA* mutant cell lines compared to the HMOsisEC10 cells, which was associated with the aggressive behavior of the benign tumor.

The experimental cell line HMOsisEC10 was developed from ovarian endometriotic epithelial cells without any evidence of cancer. Genome-wide association studies have revealed that *KRAS* and *PIK3CA* mutations with a high mutant allele frequency accounted for 38% and 29% of the endometriotic epithelium sample mutations, respectively [14]. To confirm the presence of any type of somatic mutation, we subjected the developed cell line to whole-exome sequencing analysis, which revealed no somatic or germline mutations (Table S2). Consistent with a previous report, we confirmed that the established HMOsisEC10 cell line was isolated from the portion of an ovarian cyst with no somatic mutations. Subsequently, we established two new cell lines through transfection of *KRAS* and *PIK3CA*, which are frequently mutated genes in adverse conditions and are more relevant to carcinogenic behavior.

One study has shown that driver mutations in *KRAS* and *PIK3CA* may be both the reason and result of fibrogenesis, which may increase the risk of malignant transformation from benign endometriosis [30]. The *KRAS* gene is one of the major mutated isoforms of RAS, which encodes a signaling protein that can be activated by a variety of extracellular responses and relay the signal to the downstream RAS/MAPK signaling pathways, and is associated with all forms of cancerous phenotypes, such as increased cell growth, invasion, and migration [16,31,32]. Mutations in the *PIK3CA* gene are relevant in the PI3K/AKT signaling pathway, which is involved in tumors of the brain, colorectal, breast, and other tumor types, and is associated with cell proliferation, invasion, and metastasis [33]. Previous reports found that a reduced concentration of serum in media (0.5%) decreased the growth rate of wild-type cells compared to the mutated cells, which is consistent with the results of our study. The *PIK3CA* mutant cell line had a 6–8-fold increase in migratory and invasive abilities compared to the wild-type cell line [34]. *PIK3CA* uses ATP to phosphorylate phosphatidylinositol, and mutations in *PIK3CA* were the most frequent genetic alterations of the PI3K pathway, which confers its enhanced growth and survival potential in adverse conditions [35]. The dominant nature of activated *KRAS* mutations causes aggressiveness in pancreatic cancer, and *KRAS* has a well-established role in cell proliferation, migration, and invasion [32,36,37,38]. The results of the present study and those of previous studies suggest that the immortalized *PIK3CA* and *KRAS* mutant cell lines exhibit a higher degree of cell proliferation, invasion, and migration compared to normal endometriotic epithelial cells. However, these changes were not sufficient to induce the development of cancer from endometriosis. In the present study, immortalized HMOsisEC10 cells with *KRAS* and *PIK3CA* mutations failed to develop any colonies during the soft agar assay, while the in vivo model showed insufficient metastatic properties. Case and cohort studies have shown that the risk of malignant transformation of endometriosis was 0.3–0.8% [39,40], which supports the theory that at least three sequential genetic mutations are required to convert a single normal cell into a malignant one [41]. The HMOsisEC10 *KRAS* and HMOsisEC10 *PIK3CA* mutant cell lines in our study were insufficient, despite having driver mutations and aggressive behavior, to induce cancer development [3].

By scrutinizing gene expression profiles in these cells, it was revealed that several genes were associated with their cancer-like behavior. In this study, we have established the involvement of *LOX* and *PTX3* in *KRAS* and *PIK3CA* mutated endometriotic epithelial cell migration, invasion, and proliferation-the key steps of the metastatic cascade. *LOX* gene expression is well established as a biomarker of many carcinomas and is upregulated in endometriosis tissue samples collected from humans as well as in an animal model of the disease [42,43]. In the recent past, LOX isoforms were identified as factors in the epithelial-mesenchymal transition (EMT), an event by which cancer cells gain motility and invasive mesenchymal cell properties [43]. Invasive epithelial cell lines show higher levels of LOX expression, whereas PTX3 promotes human liver cell growth [44,45]. A previous study reported that LOX cooperates with SNAIL at the protein level to facilitate the EMT and, thus, increases the invasive ability of breast cancer cell lines [46]. Another study also claimed that the overexpression of LOX promotes the proliferation, migration, and invasion of gastric cancer cell lines as well as high grade serous ovarian carcinoma [47,48]. In addition, PTX3 has pleiotropic roles in different types of cancer, such as gastric cancer, breast cancer, glioma, and prostate cancer. In hepatocellular carcinoma, several tissue studies have shown that increased PTX3 boosted cell proliferation, invasion, and migration capacity [49]. Consistent with these findings, it was revealed through microarray data in our study that *LOX* and *PTX3* were mechanistically related to increased cell proliferation, invasion, and migration. Knockdown of *LOX* and *PTX3* in different mutant cell lines led to a significant decrease in cell proliferation, migration, and invasion abilities. Silencing or inhibition of *LOX* has been reported to suppress the migration, invasion, and growth ability of esophageal squamous cell carcinoma by downregulating PIK3 harboring a *PIK3CA* mutation [44]. Conversely, knockdown of *PTX3* significantly decreased the metastatic potential of cervical cancer cells as well as the migration and invasion ability of invasive melanoma cells in vitro [49,50].

Current treatment strategies for endometriosis, including surgical removal and hormonal therapy, are often unsatisfactory. Surgical removal is a commonly used treatment for severe endometriosis, but extraction can be hazardous and painful, and some patients may have unapproachable or remote tumors. Without hormonal treatment, the tumor returns within 5 years in a relevant percentage of surgical cases [51]. In addition, some hormonal therapies can significantly reduce the level of circulating estrogen, which causes delayed conception [52].

PI3K/AKT signaling pathway was increased in ovarian endometriosis, resulting from the aberrations in the regulation of progesterone receptor (PR). These inadequate responses to progesterone receptor in endometriosis could be associated with endocrine therapy resistance. In addition, oxidative stress is also involved in progesterone resistance in endometriosis [53,54].

Furthermore, low dose estrogen progestin and visanne are often contraindicated, as they are associated with a risk of thrombosis or lipid disorder. Therefore, *LOX* and *PTX3* may be potential therapeutic targets for aggressive endometriosis. Liquid biopsies of circulating tumor DNA (ctDNA) is a more accurate non-invasive diagnostic and monitoring tool for detecting tumor-specific mutations. Examination of cfDNA load with *KRAS* and *PIK3CA* mutation allows for the prediction of the abnormalities at an early stage, which can improve treatment outcomes. Silencing *LOX* and *PTX3* expression using small molecules is a potential treatment strategy for reducing cell migration, invasion, and proliferation, especially in deep infiltrating endometriosis with *KRAS* and *PIK3CA* mutations.

## 4. Materials and Methods

### 4.1. Ethical Approval

This study was approved by the institutional ethics review board of Shimane Medical University, Izumo, Shimane, Japan (IRB No. 20070305-1 and No. 20070305-2, 5 March 2007).

### 4.2. Isolation and Purification of the Tissue Samples

Human endometriotic tissue samples were obtained from ovarian endometriotic cysts collected from three different patients. Stromal cells were identified in one sample, and hence, it was discarded. Immortalized endometriotic epithelial cells were generated successfully from one of the remaining two samples with a success rate of 33%. This immortalized endometriotic epithelial cell line was used in our study. The 48-year-old patient from whom endometriotic epithelial cells were collected successfully underwent a laparoscopic ovarian cystectomy as treatment for ovarian endometriosis. Briefly, the tissue samples were gently but thoroughly washed with sterile phosphate-buffered saline (PBS) to remove blood contamination. Next, the samples were dissected into small pieces (1–2 mm^3^) using sterile scissors and digested with collagenase type 3 (Washington Biochemical Corp. Lakewood, NJ, USA) in a shaking water bath at 37 °C for 60 min. To separate the epithelial glands from the stromal cells and debris, serial filtration through narrow gauge sieves was performed, whereby debris was removed using 100-micrometer aperture sieves, and epithelial glands were retained on 40-micrometer sieves. Individual glands were visualized under a microscope, collected into a micro centrifuge tube, and cultured in 24-well dishes for subsequent gene transfection using viral vectors. The patient included in this study provided written informed consent for the use of her clinical and pathological specimens.

### 4.3. Vector Construction and Cell Transfection

To generate immortalized cells, previously collected normal endometriotic cells were transduced with *hTERT*, *cyclin D1*, and mutant *CDK4* (CDK4^R24C^: an inhibitor-resistant form of CDK4) via lentivirus-mediated gene transfer using the Gateway system that has been described previously [55,56]. The construction of the recombinant lentiviruses with the help of the vesicular stomatitis virus G glycoprotein has also been described previously [57]. cDNAs encoding mutant *KRAS (KRAS*^v12^*)* and *PIK3CA* were gifted to us by Dr. Goto (Aichi Cancer Research Institute, Nagoya, Japan). *KRAS* and *PIK3CA* mutant-overexpressing cells were established by lentivirus vector infection of the human *KRAS* and *PIK3CA* expression vectors pCMSCV-EM7bsd-*^KRAS^* and pCMSCV-EM7bsd-*^PIK3CA^*. The cell lines were named as HMOsisEC10, HMOsisEC10 *KRAS* mutant, and HMOsisEC10 *PIK3CA* mutant cell lines.

### 4.4. Cell Lines and Cell Culture

Immortalized human ovarian endometriotic epithelial cell lines HMOsisEC10, mutant HMOsisEC10 *KRAS*, and HMOsisEC10 *PIK3CA* were established from ovarian endometriotic epithelial cells. All cell lines were grown in F-medium supplemented with 5% fetal bovine serum (FBS; 10437-028, Gibco) and 1% penicillin-streptomycin solution (P4333, Sigma-Aldrich) and maintained at 37 °C in a humidified atmosphere with 5% CO2. Images of the cell lines are presented in Figure S1.

### 4.5. Western Blot Analysis

Cell lysates were prepared by disrupting cell pellets in lysis buffer. Samples were prepared by adding LDS buffer and sample-reducing buffer, heated at 70 °C for 10 min, cooled on ice for 1 min, and then centrifuged at 150 rpm for 5 min. Samples were then separated using SDS-PAGE (Invitrogen) and transferred onto PVDF membranes using Bio-Rad semi-dry trans blotters. Membranes were blocked with Licor blocking buffer (LI-COR, Lincoln, NE, USA) for 1 h at room temperature and incubated with primary antibody (Table S3) diluted in LI-COR blocking buffer containing 0.1% Tween. The membrane was kept overnight on a shaker at 4 °C. The membranes were washed four times for 5 min in TBST and then probed with goat anti-mouse or goat anti-rabbit secondary antisera labeled with IRDye 670 or 800 CW diluted in LI-COR blocking buffer containing 0.1% Tween and 0.01% SDS was kept at room temperature on a shaker for 1 h covered with aluminum foil. The probe membranes were washed four times for 5 min in TBST, finally added to TBS, and imaged using a LI-COR Odyssey scanner. Boxes were manually drawn around each band of interest, and near-infrared fluorescent values for raw intensity, with intra-lane background subtracted, were obtained using Odyssey 3.0 analytical software (LI-COR).

### 4.6. Immunocytochemistry

Cells were cultured on a chamber slide (Sumilon cell desk LF1) in 24-well plates (50,000 cells per well). After 24 h of incubation, the plate was removed from the incubator and washed three times with PBS (0.5 mL). Cells were then fixed with 4% formalin for 10 min at room temperature and washed once with PBS. Samples were permeabilized with 0.1–0.5% Triton X-100 in PBS for 10 min and then washed with PBS three times (2 min, once; 5 min, twice) on a shaker. Samples were incubated in 10% normal goat serum for 1 h at room temperature. After blocking, the primary antibody (dilution 1:50) was added, incubated at 4 °C overnight, and then washed with PBS three times (2 min, once; 5 min, twice) on a shaker. The secondary antibody was added, incubated for 1 h covered with aluminum foil, and then washed with PBS three times (2 min, once; 5 min, twice) on a shaker. The cells were stained with DAPI, and the slide was sealed using nail polish enamel and viewed under a microscope.

### 4.7. Short Tandem Repeat (STR) Analysis

DNA extraction was performed on three passages of the HMOsisEC10 line using a DNeasy^®^ Blood and Tissue Kit (QIAGEN, Hilden, Germany), following manufacturer protocol. Eight human STR loci (Table S5) were amplified in the ABI PRISM 310 genetic analyzer with 10 ng of template DNA. Next, PCR was conducted in a BIO RAD T100^TM^ thermal cycler. Amplicons were visualized with 2% agarose gel electrophoresis and quantified using a spectrophotometer (NanoDrop 2000; Thermo Fisher Scientific, Waltham, MA, USA) to ensure accuracy and concentration uniformity. Amplicon aliquots (1 µL) were placed in 0.5 mL sample tubes with 10 µL deionized formamide and 0.5 µL size-standard Gene Scan^TM^ 500 (Applied Biosystems). They were denatured at 95 °C for 5 min, instantly cooled on ice, and subjected to another electrophoresis in an ABI PRISM 310 genetic analyzer. Data were analyzed with ABI Genemapper v4.1 (Applied Biosystems).

### 4.8. Simulated Wound Healing Assay to Assess Cell Motility

Cells were seeded onto 6-well plates at a density of 1 × 10^6^ cells per well and grown to a confluent monolayer. An acellular area was created by scraping the cell surface using a 200-microliter pipette tip (time 0). Non-attached cells were removed by gentle washes using the culture medium. The rate of wound closure was measured by monitoring the wound healing process for 24 h. The cells that migrated to the wound area were counted 24 h after scraping. The individual cells in the monolayer defect were quantified as an average from multiple fields (at least five) at 200× magnification.

### 4.9. Matrigel Invasion Assay

The invasion assay was performed using a Corning Bio Coat Matrigel Invasion Chamber (Discovery Labware Inc., Bedford, MA, USA) with an 8-micron pore size. The chamber was removed from the refrigerator and 500 µL of serum-free medium was added to the upper chamber along with the bottom chamber. The chambers were kept in a humidified tissue culture incubator at 37 °C with 5% CO_2_ for 1–2 h. The serum-free medium was carefully removed from both the upper and bottom chambers. Cells were seeded at a density of 25,000/350 µL in serum-free medium into the upper chamber. The lower compartment contained 900 µL of F-medium containing 20% FBS as a chemoattractant. After 24 h of incubation at 37 °C under 5% CO_2_, the chambers were removed from the incubator; the medium was removed from both the chambers followed by washing twice with sterile PBS and the addition of 3.7% paraformaldehyde to both the upper and lower chambers for 2 min. Subsequently, the wells were washed twice with PBS, and methanol was added to both the upper and lower chambers for 20 min. After treatment with methanol, the wells were washed twice with PBS, and 5% Giemsa stain was added to both the upper and lower chambers for 15 min. Subsequently, the wells were washed twice with PBS followed by careful removal of the uninvaded cells with a cotton swab. Cells migrating through the membrane and cells invading the Matrigel were counted in 16 non-overlapping 200× fields under a light microscope.

### 4.10. Cell Proliferation Assay

Cells were seeded in a 96-well plate at a density of 3000 cells per well. Cell number was determined indirectly using an MTT assay [58]. The data were calculated as the mean ± standard deviation (SD) of triplicate replications.

### 4.11. Anchorage Independent Assay

A total of 10,000 cells were seeded into 24-well plates containing a top layer of 0.33% noble agar in 2× DMEM supplemented with 5% FBS and a bottom layer of 0.5% base agar in 2× DMEM supplemented with 5% FBS. After solidification of the gel, each well was covered with 1 mL of the culture medium and incubated at 37 °C. Colonies larger than 0.05 mm in diameter were counted after incubation for 2 weeks.

### 4.12. Nude Mice Xenograft

We injected each cell line (2.5 × 10^7^ cells/mL) into the subcutaneous tissues of 4-week-old athymic BLAB/c nu/nu mice (Charles River Japan INC., Kanagawa, Japan). Four mice were used for each experimental group. Tumor growth was monitored weekly for two months or until the mice became moribund. All animal experiments were performed in accordance with the regulations of the institutional ethical commission.

### 4.13. Whole-Exome Sequencing

Whole-exome sequencing was performed on the HMOsisEC10 cell line to determine the presence of any protein-coding mutations; the genome sequencing methods used have been previously described [59]. Briefly, quality control was assessed by generating a DNA integrity number using the Agilent 2000 TapeStation (Agilent Technologies, Santa Clara, CA, USA) prior to targeted amplicon whole-exome sequencing with the Illumina MiSeq sequencing platform (Illumina, San Diego, CA, USA). The lowest quality of DNA had a DIN score over 3.1, whereas the lowest DNA concentration was 50 ng. The sequencing data were analyzed using the GenomeJack bioinformatics pipeline (Mitsubishi Space Software, Tokyo, Japan) through various steps, including sequence alignment, variant calling, variant filtering, variant annotation, and variant prioritization to ensure accurate reporting of analytic sensitivity and specificity. We identified no cancer-specific alterations in the somatic genes, including single-nucleotide variations, insertions/deletions, and gene copy-number alterations. These results were used to determine the tumor mutational burden.

### 4.14. Microarray Analysis

Microarray analysis was performed at Takara Bio Incorporation (Tokyo, Japan) using an Agilent Sureprint G3 Human GE 8 × 60 K microarray (Agilent Technologies, Palo Alto, CA, USA) containing 42,545 probe sets, according to the manufacturer’s protocol. Briefly, RNA for cDNA microarray analysis was purified using RNeasy column purification (QIAGEN). Accuracy and concentration uniformity were confirmed using a Nano Drop ND-2000 spectrophotometer. Total RNA was labeled, amplified, hybridized, and scanned using the standard Agilent protocol. HMOsisEC10, HMOsisEC10 *KRAS,* and HMOsisEC10 *PIK3CA* samples were run on different ID columns including Agilent probes. Raw data were analyzed in feature extraction software (Agilent Technologies) using default parameters to obtain detrended processed signal intensities. The signal intensity data were normalized by the global scaling method. Duplicated probes on the array were treated independently during normalization and statistical analyses. To avoid extreme fold-change artifacts, negative or low-intensity signals less than 10 were corrected to 10. The optimal selected gene probes were used to score the correlation of all muted cell samples with the *KRAS* and *PI3KCA* gene signature. Depending on whether the gene signature score exceeded a predefined threshold, a sample was classified as an activated oncogenic or wild-type-like.

### 4.15. siRNA Transfection

RNA interference and siRNA preparation were performed over 48 h. Effects of siRNA (*LOX* and *PTX3*) (Santa Cruz Biotechnology) were compared with those of a control siRNA and untreated cells. Cells were plated into 6-well plates for the migration assay and allowed to grow to a confluent monolayer before transfection. Transfection methods were as follows: mixing Opti-MEM^®^ medium 150 µL and Lipofectamine^®^ RANIMAX 9 µL, and mixing Opti-MEM^®^ medium 150 µL and 6 µL (20 nm) siRNA (*LOX* and/or *PTX3*). Then, diluent siRNA was added to the diluent Lipofectamine^®^ RANIMAX reagent (1:1) and incubated for 5 min at room temperature. We subsequently added 250 µL of the siRNA mix into each well. After 48 h, scratches were made using a 200-microliter pipette. For the invasion assay, 25,000 cells were plated into 24-well plates mixing Opti-MEM^®^ medium 50 µL and Lipofectamine^®^ RANIMAX 3 µL, and 50 µL of Opti-MEM^®^ medium and 2 µL (20 nm) siRNA (*LOX* and/or *PTX3*), followed by incubation for 5 min at room temperature. Next, 50 µL of siRNA mix was added into each well. After 48 h of transfection, cells were transferred onto a Matrigel-coated upper chamber, and 20% chemoattractant was added to the bottom chamber. After 24 h, we checked for cell invasion. For the proliferation assay, the cells were transfected with siRNA (*LOX* and/or *PTX3*) in 96-well plates at 20 nM and the results were obtained on days 0, 2, and 3. To avoid off-target effects, different siRNAs (*LOX* and *PTX3*; Ambion Life Technologies) were also used to perform the migration, invasion, and proliferation assays. The sequences of the siRNAs (*LOX* and *PTX3*) are listed in Table S4.

### 4.16. Reverse Transcriptase Polymerase Chain Reaction (RT-PCR)

The cell pellet was homogenized using QIAGEN buffer RLT (QIAGEN GmbH, QIAGEN, Hilden, Germany). Total RNA was isolated using a standard protocol (Qiagen N.V) according to the manufacturer’s instructions. The RNA quantity was measured by spectrophotometry using a NanoDrop ND-1000 (NanoDrop Technologies, Wilmington, DE, USA). The reverse transcriptase polymerase chain reaction (RT-PCR) was performed using the Applied Biosystems SYBR Green master mix kit, purchased from Thermo Fisher Scientific, Inc. The primer sequences used for RT-PCR are included in Table 1. Standard cycling conditions were used: 95 °C for 30 s, 95 °C for 5 s, 60 °C for 30 s followed by 40 cycles at 95 °C for 15 s, and 60 °C for 30 s, then 95 °C for 15 s. Gene expression levels were standardized to the levels of *GAPDH* using the 2^–ΔΔCt^ method. The experiment was independently repeated at least three times.

## 5. Conclusions

In summary, by using the immortalized human ovarian endometriotic cell line HMOsisEC10, we have established two new mutant cell lines, HMOsisEC10 *KRAS* and HMOsisEC10 *PIK3CA,* through in vitro vector construction and cell transfection. Mutant cells exhibited a significant increase in proliferation, migration, and invasion potential. Microarray gene-expression analysis revealed several genes that demonstrated correlations with *KRAS* or *PIK3CA* invasion-specific gene signatures, which played essential roles in activating the endometriosis progression pathways. We showed that LOX and PTX3 were the main downstream targets of the *KRAS* and *PIK3CA* mutant endometriotic epithelial cells. Thus, we speculate that the inhibition of LOX and PTX3 could provide a better treatment for ovarian endometriosis. Furthermore, this experimental model may be the key to attaining a better understanding of the functional and molecular pathogenesis of endometriosis and might provide a novel therapeutic target to reduce the further incidence of endometriosis.

## Figures and Tables

**Table 1:** List of RT-PCR primer sequences for validation of microarray data.

Name of Primer	Type	Sequence (5′-3′)
*LOX*	Forward	TGCCAGTGGATTGATATTAC
	Reverse	TACGGTGAAATTGTGCAGCC
*PTX3*	Forward	GCATCTCCTTGCGATTCTGTT
	Reverse	CATTCCGAGTGCTCCTGACC
*GAPDH*	Forward	ACGGGAAGCTTGTCATCAAT
	Reverse	TGGACTCCACGACGTACTCA

## Data Availability

The data that support the finding of this study are available from the corresponding author upon responsible request.

## Supplementary Materials

The following are available online at https://www.mdpi.com/article/10.3390/cancers13133174/s1, Figure S1: Morphological characteristics of immortalized endometriotic epithelial cells: HMOsisEC10, HMOsisEC10 KRAS, and HMOsisEC10 PIK3CA. Figure S2: Western blot (left panel) and immunocytochemical analyses (right panel) of pan-cytokeratin expression in immortalized HMOsis EC10 (WT) cells. Figure S3: Western blot analysis of pan-cytokeratin, pan-AKT, phospho-AKT, pan-MAPK and phospho-MAPK expression in different transfectants. (A) Immortalized endometriotic epithelial cells (HMOsisEC10), (B) HMOsis EC10 KRAS cells, and (C) HMOsisEC10 PIK3CA cells. Figure S4: Short tandem repeat (STR) analysis of HMOsisEC10 immortal cells. Comparison of genotyping results in adjacent HMOsisEC10 cells at passages 2, 13, and 22 with eight STR loci (TPOX, CSF1P0, VWA, FGA, D3S1359, D18S51, D8S315, and SE33). All eight loci were amplified and demonstrated to have identical genotypes, indicating that there was no cross-contamination between the different cell passages. Figure S5: Anchorage independent assay. No colony formation was observed in the mutant cell lines HMOsisEC10 KRAS (left) and HMOsisEC10 PIK3CA (middle). Image was acquired 30 days following passage. The SKOV3 cell line (positive control) showed colony formation after 30 days of passage on the agar medium (right). Figure S6: In vivo inoculation of HMOsisEC10 KRAS (left) and HMOsisEC10 PIK3CA mutant cells (middle) into mice; the photograph was captured 60 days after inoculation. No tumor development was observed in the mice inoculated with the mutant cell lines. However, tumor development was observed in the mouse inoculated with the SKOV3 cell line (right) as a positive control. Figure S7: In the exome sequencing result, copy number alteration (CNA) plot shows DNA from both parental lines (maternal and paternal) has no significant variation/mutation present. Figure S8: Validation of microarray data using RT-PCR. The mean fold changes in the mRNA expression level of *LOX* and *PTX3* are shown on the *y*-axis. For both LOX and PTX3 mRNAs, the microarray and RT-PCR results are consistent with those in HMOsisEc10 KRAS and HMOsisEC10 PIK3CA mutant cell lines. Figure S9: Cell migration assay. Migration abilities of mutant cell lines (HMOsisEC10 KRAS and HMOsisEC10 PIK3CA) measured using a scratch wound healing assay after knockdown with *LOX* (A1, A2) and *PTX3* siRNA (B1, B2). The numbers of migrated cells were significantly lower in siRNA-treated cells compared to the control siRNA and untreated cells. Matrigel invasion assay; siRNA knockdown of *LOX* (C1, C2) and *PTX3* (D1, D2) showed a significantly lower invasion capacity than control siRNA and untreated cells. Cell proliferation assay. Treatment with *LOX* (E1, E2) and *PTX3* siRNA (F1, F2) significantly reduced cell proliferation ability relative to the control siRNA and untreated cells. ** *p* < 0.01 and * *p* < 0.05. The error bars indicate standard deviation. Table S1: Description of primary antibodies. Table S2: Whole-exome sequencing result of HMOsisEC10 cells. Table S3: Gene expression level of RhoA, TNF-α, and SRF. Table S4: Sequences of siRNA LOX and siRNA PTX3. Table S5: Primers for STR amplicon and chromosomal location.

## Abbreviations

*KRAS:*	Kristen Rat Sarcoma
*PIK3CA*	Phosphatidylinositol-4,5-Bisphosphate 3-Kinase Catalytic Subunit Alpha
LOX	lysyl oxidase
PTX3	Pentraxin3
RT-PCR	Reverse Transcriptase Polymerase Chain Reaction
RhoA	Ras homolog family member A,
TNF-α	Tumor Necrotic Factor- α
SRF	Serum Response Factor
hTERT	humans Telomerase Reverse Transcriptase
CDK4	Cyclin Dependent Kinase 4
STR	Short Tandem Repeat
siRNA	small interfering RNA
