# Supplementary material for: Establishment of a Novel In Vitro Model of Endometriosis with Oncogenic KRAS and PIK3CA Mutations for Understanding the Underlying Biology and Molecular Pathogenesis
Source: Cancers (Basel). 2021 Jun 25;13(13):3174. doi: 10.3390/cancers13133174 (PMC8269352; doi:10.3390/cancers13133174)
Supplement: Supplementary file 1 [file cancers-13-03174-s001.zip › cancers-1195416-supplementary.pdf]

Supplementary Materials:

Establishment of a Novel In Vitro Model of Endometriosis with Oncogenic *KRAS* and *PIK3CA* Mutations for Understanding the Underlying Biology and Molecular Pathogenesis

Hossain Mohammad Mahmud, Kentaro Nakayama, Kamrunnahar Shanta, Sultana Razia, Masako Ishikawa, Tomoka Ishibashi, Hitomi Yamashita, Seiya Sato, Kouji Iida, Kosuke Kanno, Noriyoshi Ishikawa, Tohru Kiyono and Satoru Kyo

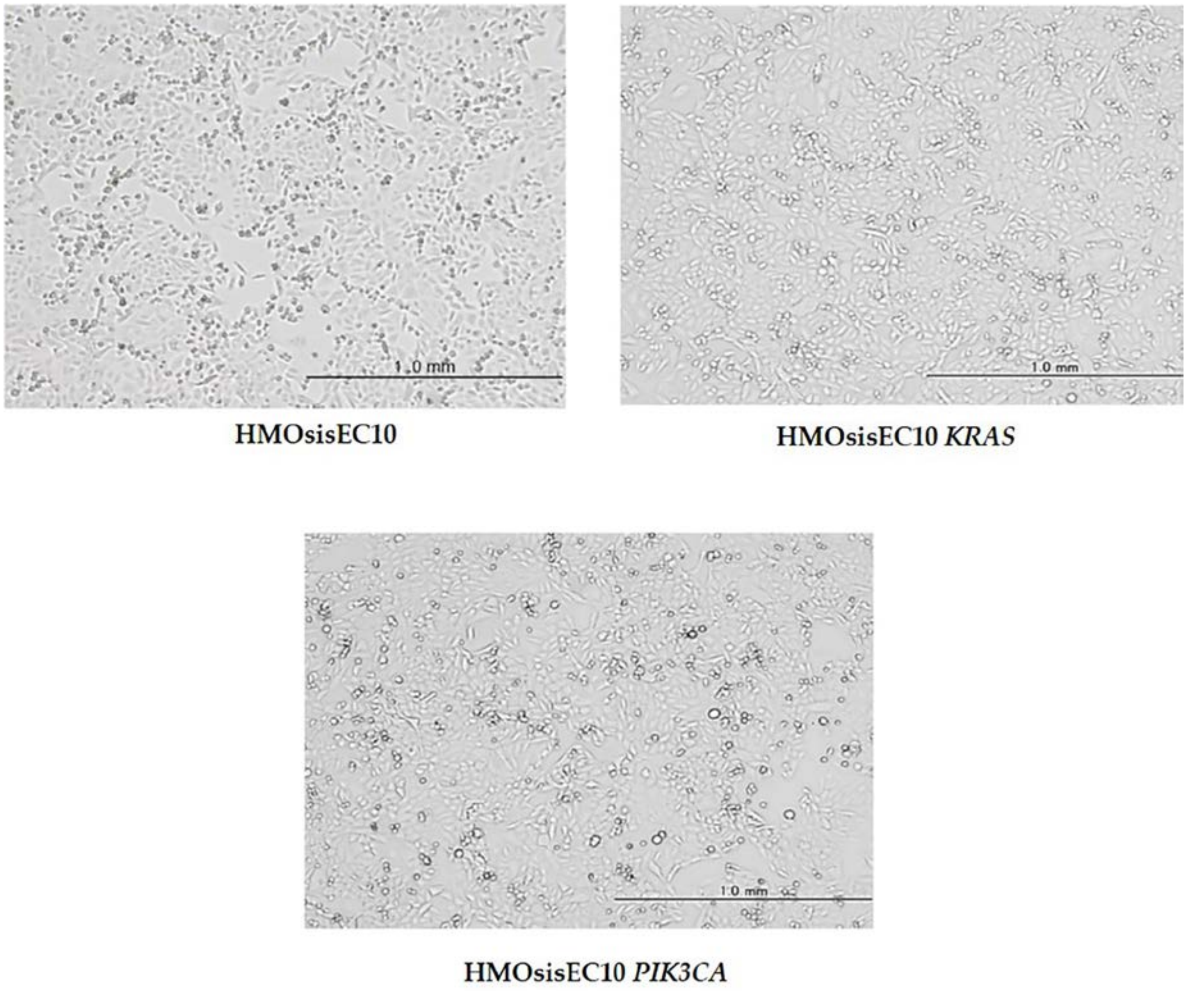

Figure S1: Morphologic characteristic of immortalized endometriotic epithelial cells HMOsisEC10, HMOsisEC10 *KRAS* and HMOsisEC10 *PIK3CA*.

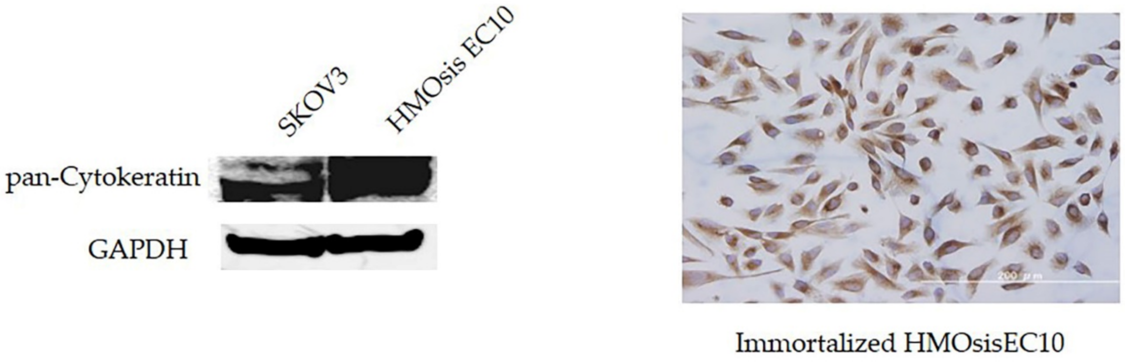

Figure S2: Western blot (left panel) and immunocytochemical analysis (right panel) of pan-cytokeratin expression in immortalized HMOsis EC10 (WT) cells.

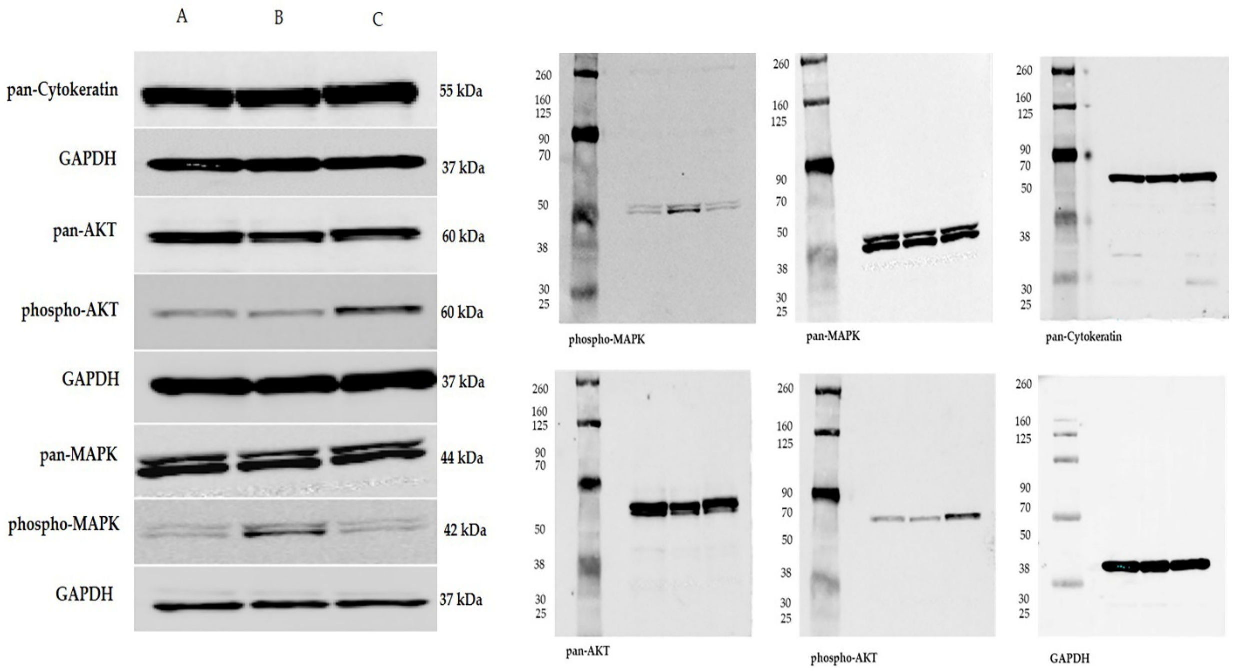

Figure S3: Western blot analysis of pan-Cytokeratin, pan-AKT, phospho-AKT, pan-MAPK and phospho-MAPK expression in different transfectants. (A) Immortalized endometriotic epithelial cells (HMOsisEC10), (B) HMOsis EC10 *KRAS* and (C) HMOsisEC10 *PIK3CA*.

Table S1: Description of primary antibodies.

Antibody	Manufacturer	Dilution	MW (kDa)
Anti-pan-Cytokeratin	Shanta Cruz Biotechnology (sc-8081)	1:200 (WB)	50
		1:50 (IHC)	
Anti-AKT (pan)	Cell signaling (# 4691)	1:1000 (WB)	60
Anti-p44/42 MAPK (Erk1/2)	Cell signaling (# 4695)	1:1000 (WB)	42,44
Anti-phospho-AKT (ser473)	Cell signaling (# 4060)	1:2000 (WB)	60
Anti-phospho-p44/42 MAPK (Erk1/2) (Thr202/Tyr204)	Cell signaling (# 4370)	1:2000 (WB)	42,44
GAPDH	Cell signaling (14C10)	1:1000	37

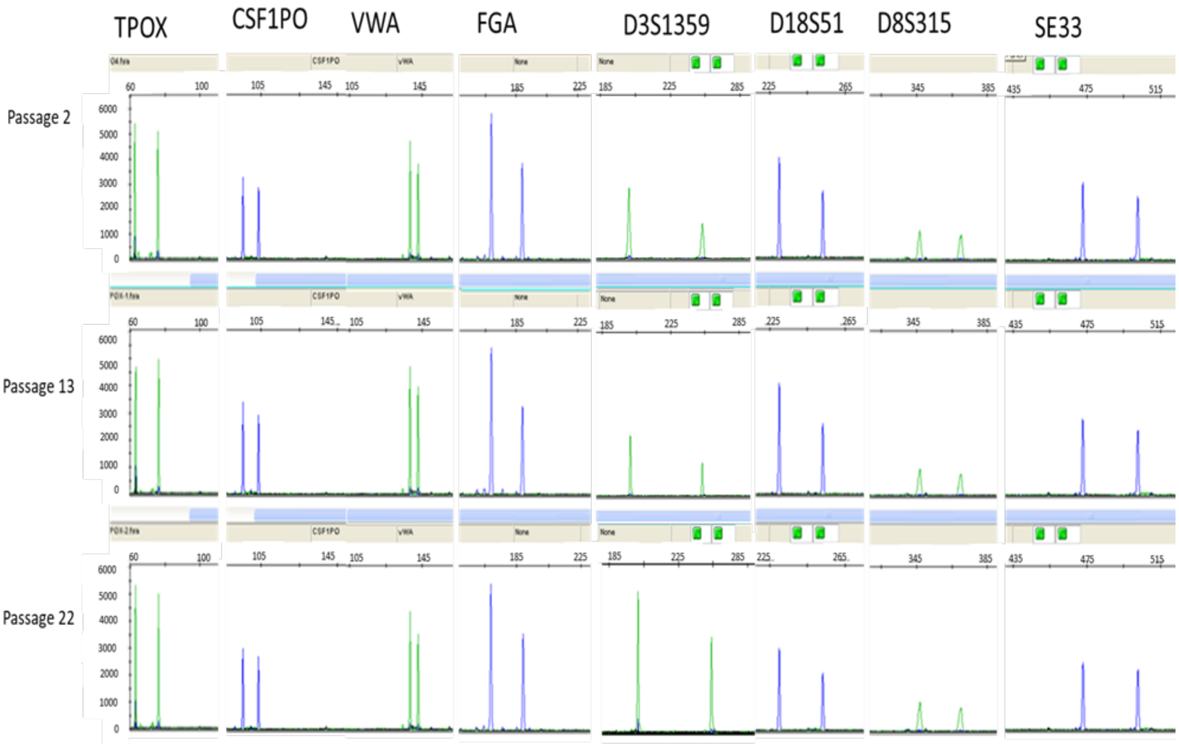

Figure S4: Short tandem repeat (STR) analysis of HMOsisEC10 immortal cells. Comparison of genotyping results in adjacent HMOsisEC10 cells at passages 2, 13, and 22 with eight STR loci (TPOX, CSF1P0, VWA, FGA, D3S1359, D18S51, D8S315, and SE33). All eight loci were amplified and demonstrated to have identical genotypes, indicating that there was no cross-contamination between the different cell passages.

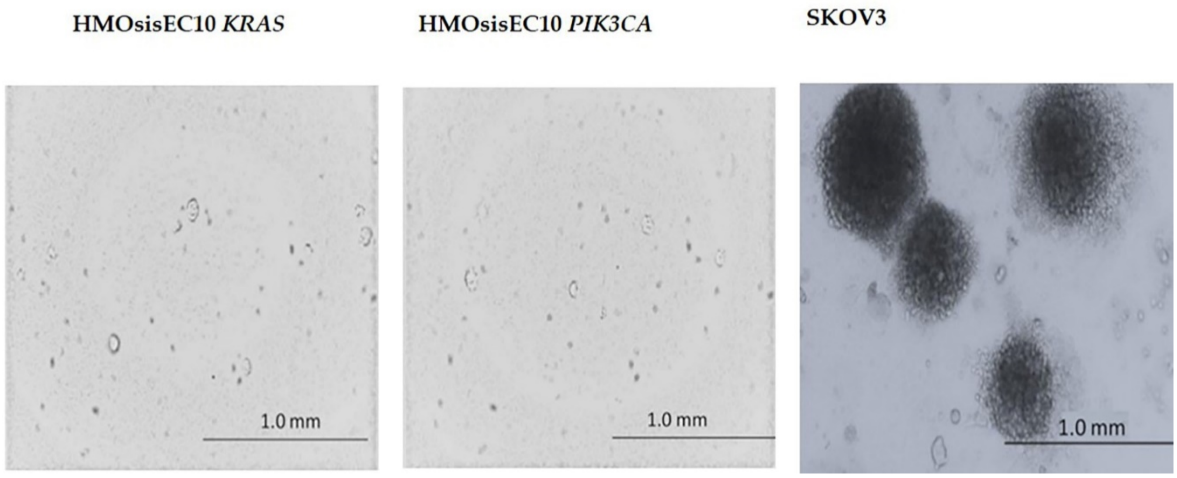

Figure S5: Anchorage independent assay; No colony formation was observed in the mutant cell lines HMOsisEC10 *KRAS* (left) and HMOsisEC10 *PIK3CA* mutant cells(middle). Image was taken 30 days post passage. The SKOV3 cell line (positive control) showed colony formation after 30 days of passage on the agar medium (right).

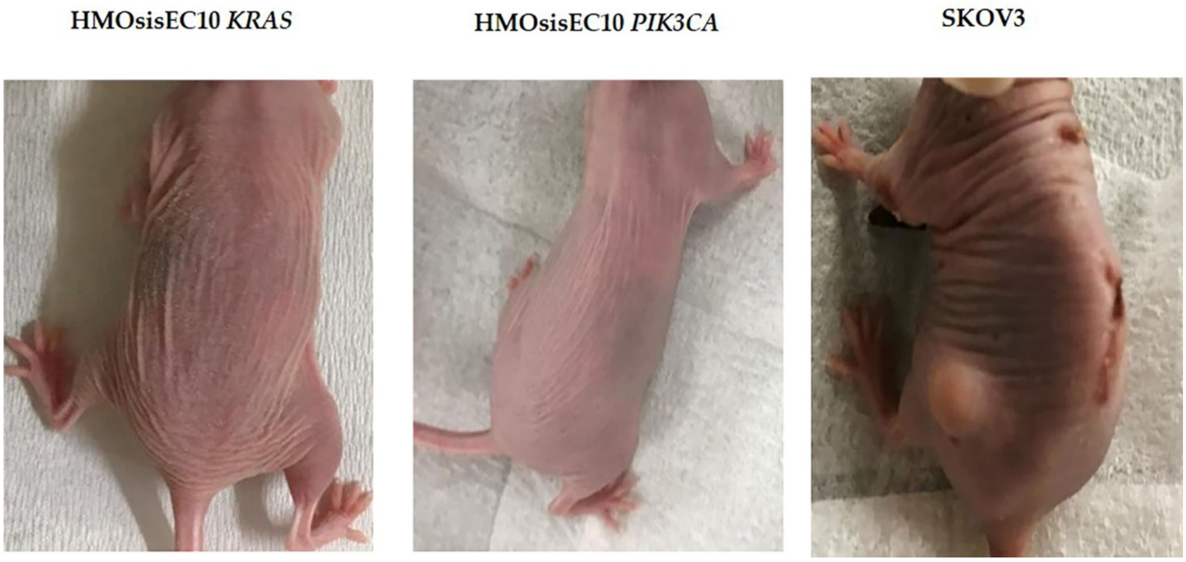

Figure S6: *In vivo* inoculation of HMOsisEC10 *KRAS* (left) and HMOsisEC10 *PIK3CA* mutant cells (middle) into mice; the photo was taken 60 days post inoculation. No tumor development was observed in the mice inoculated with the mutant cell lines. However, tumor development was observed in the mouse inoculated with the SKOV3 cells line (right) as a positive control.

Table S2: Whole exome sequencing result of HMOsisEC10.

Mutation type	Status
dMMR (Deficient Mismatch Repair)	No mutation
HRD (Homologous Recombination Deficiency) r	No mutation
OG (Oncogene)	No mutation
TSG (Tumor Suppressor Gene)	No mutation
Other gene mutation	No mutation

Note: The above-mentioned table showed that the result of whole exome sequencing reveals there were no inherent or acquired mutation present in the wild type cell line.

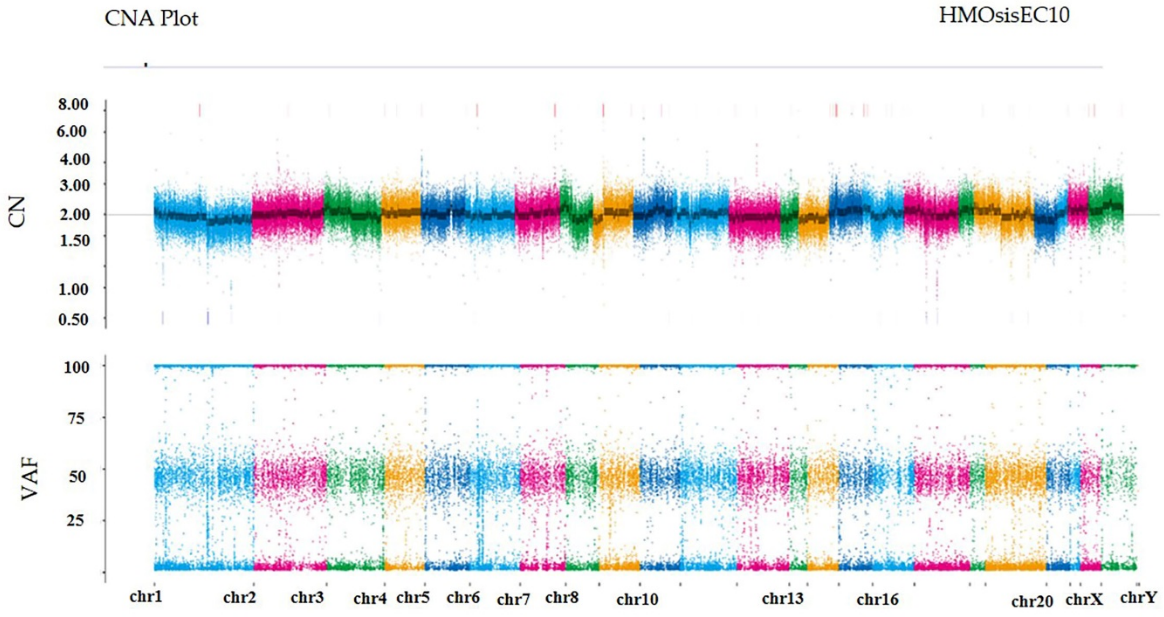

Figure S7: In the exome sequencing result CNA (Copy Number Alteration) plot shows DNA from both parental (maternal and paternal) has no significant variation/mutation present.

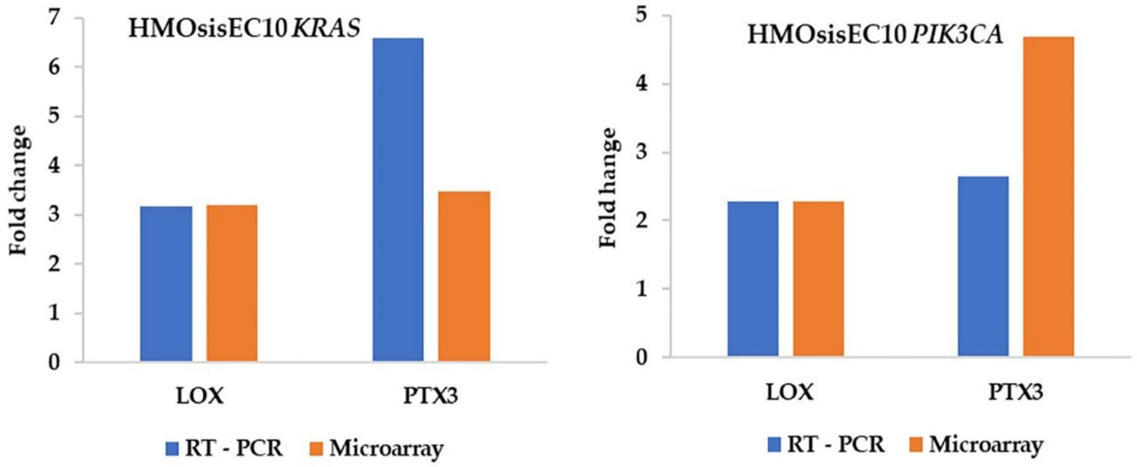

Figure S8: Validation of microarray data using RT-PCR; The mean fold change of the mRNA expression level of *LOX* and *PTX3* were shown on the y-axis. For both *LOX* and *PTX3* mRNAs, the microarray and RT-PCR results are consistent in HMOsisEc10 *KRAS* and HMOsisEC10 *PIK3CA* mutant cell line.

Table S3: Gene Expression level of RhoA, TNF-*α* and SRF.

Gene Name	HMOsisEC10	HMOsisEC10 *PIK3CA*	HMOsisEC10 *KRAS*
**RhoA**			
**TNF-*α***			
**SRF**			

Note: In the table microarray result revealed Ras homolog family member A (RhoA), tumor necrosis factor- *α* (TNF-*α*) and serum response factor (SRF) genes showed gray color in wild type HMOsisEC10 indicate no change in expression, on the other hand HMOsisEC10 *PIK3CA* and HMOsisEC10 *KRAS* mutant cells showed red color indicate high expression.

Table S4: Sequences of siRNA *LOX* and siRNA *PTX3*.

**Shanta Cruz Biotechnology**		
Name of gene	siRNA sequence	
LOX	Sense	GACAACCCUUAUUACAACUtt
	Antisense	AGUUGUAAUAAGGGUUGUCtt
PTX3	Sense	CAAAGCCACAGAUGUAUUAtt
	Antisense	UAAUACAUCUGUGGCUUUGtt
**Ambion life technologies**		
LOX	Sense	GCACAGUUGUCAUCAACAYtt
	Antisense	AUGUUGAUGACAACUGUGCca
PTX3	Sense	GGAUAGUGUUCUUAGCAAUtt
	Antisense	AUUGCUAAGAACACUAUCCca

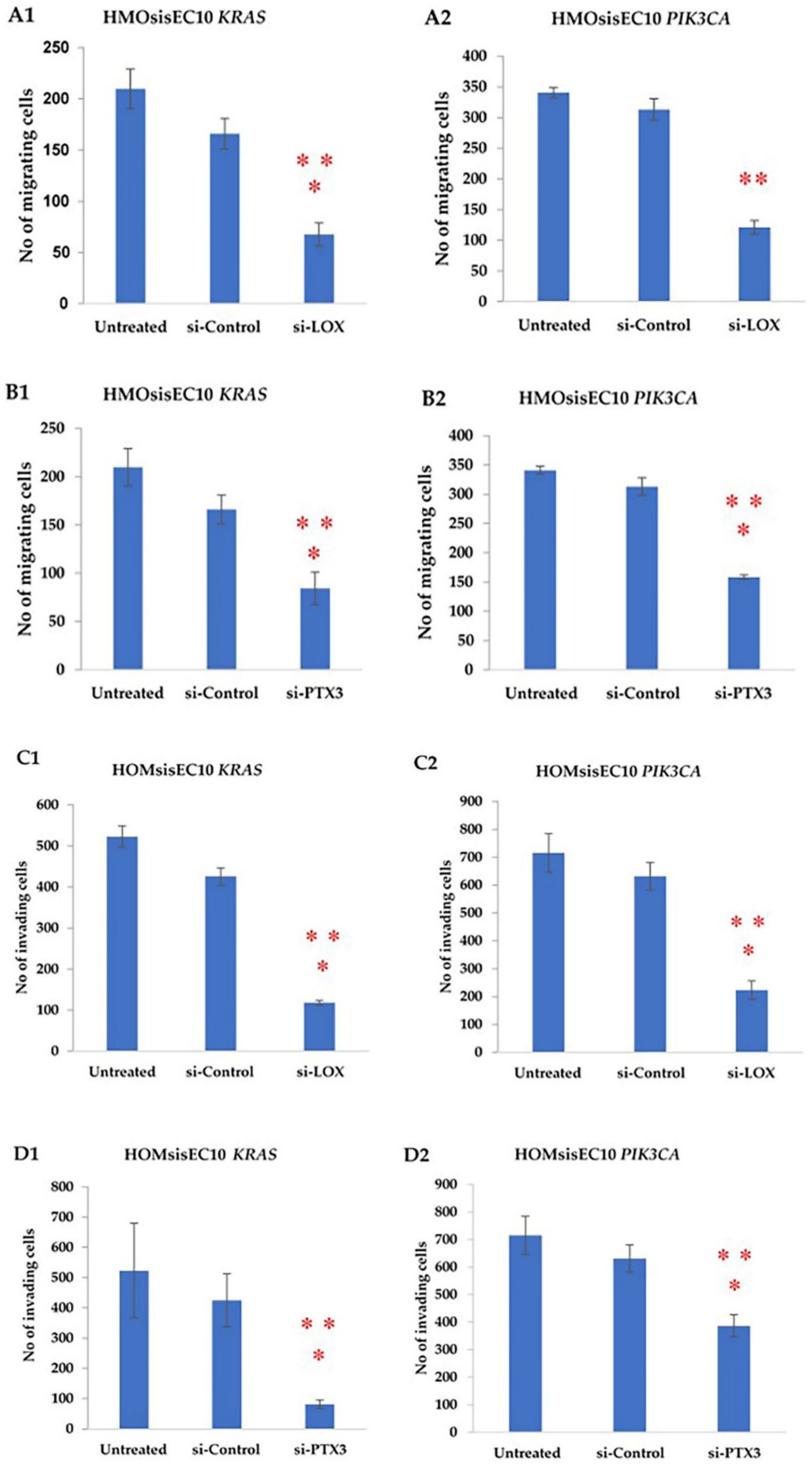
A1
HMOsisEC10 KRAS
No of migrating cells
Untreated
si-Control
si-LOX
A2
HMOsisEC10 PIK3CA
No of migrating cells
Untreated
si-Control
si-LOX
B1
HMOsisEC10 KRAS
No of migrating cells
Untreated
si-Control
si-PTX3
B2
HMOsisEC10 PIK3CA
No of migrating cells
Untreated
si-Control
si-PTX3
C1
HOMsisEC10 KRAS
No of invading cells
Untreated
si-Control
si-LOX
C2
HOMsisEC10 PIK3CA
No of invading cells
Untreated
si-Control
si-LOX
D1
HOMsisEC10 KRAS
No of invading cells
Untreated
si-Control
si-PTX3
D2
HOMsisEC10 PIK3CA
No of invading cells
Untreated
si-Control
si-PTX3

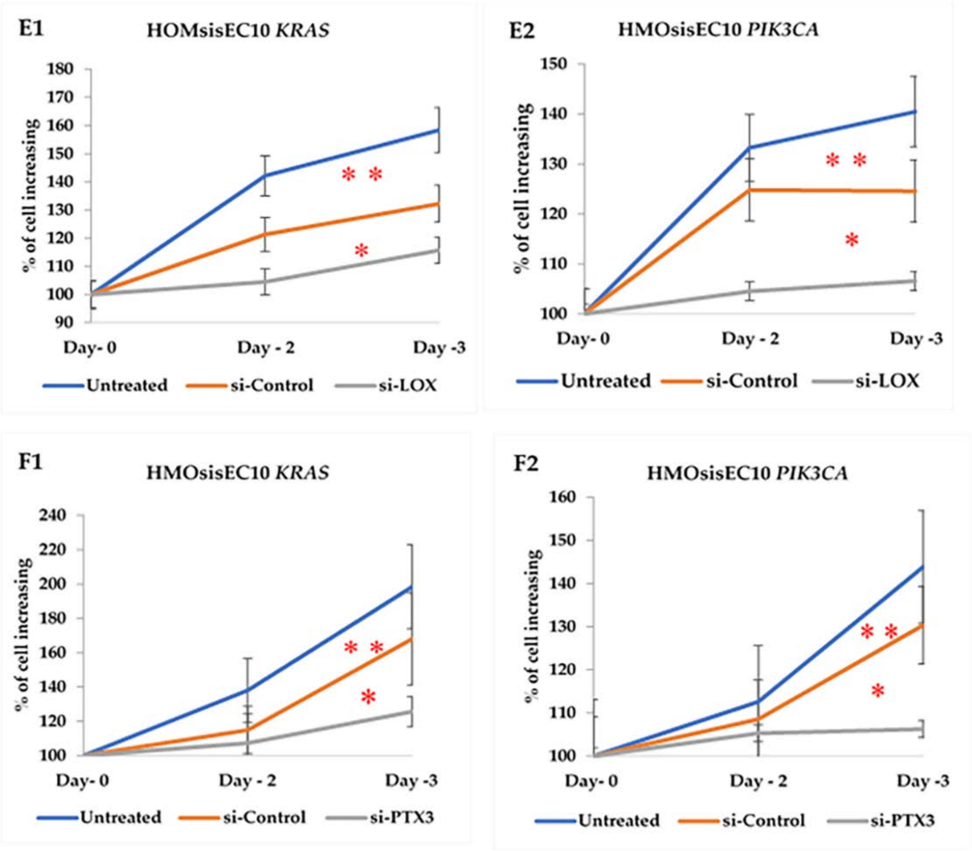

Figure S9: Cell migration assay; Migration abilities of mutant cell lines (HMOsisEC10 *KRAS* and HMOsisEC10 *PIK3CA)* are measured using a scratch wound healing assay after knockdown with *LOX* (A1, A2) and *PTX3* siRNA (B1, B2). The numbers of migrated cells were significantly lower in siRNA treated cells compared to the control siRNA and untreated cells. **Matrigel Invasion assay;** siRNA knockdown of *LOX* (C1, C2) and *PTX3* (D1, D2) showed a significantly lower invasion capacity than control siRNA and untreated cells. **Cell proliferation assay;** Treatment with *LOX* (E1, E2) and *PTX3* siRNA (F1, F2) significantly reduced cell proliferation ability relative to the control siRNA and untreated cells. **P<0.01 and *P<0.05. The error bars indicate standard deviation.

Table S5: Primers for STR amplicon and chromosomal location

Sl #	STR marker	GenBank accession	Size (BP)	Chromosomal location	Primer design	Dye level
1	*TPOX*	M68651	61–101	2pter-p24	F: CTTAGGGAACCCTCACTG<br>R: GCAGCGTTTATTTGCCCAA	JOE (G)
2	*CSF1PO*	X14720	75–111	5q33.3-q34	F: ACTGCCTTCATAGATAGAAGAT<br>R: GCCCTGTTCTAAGTACTTCCT	FAM (B)
3	*VWA*	M25858	121–173	2pter-p12	F: TCAGTATGTGACTTGGATTGA<br>R: GTAGGTTAGATAGAGATAGGACAGA	NED (Y)
4	*FGA*	M64982	151–293	4q28	F: CTCACAGATTAAACTGTAACCA<br>R: TTGTCTGTAATTGCCAGC	FAM (B)
5	*D3S1359*	M0079	213-257	3p	F: ATGCTAAGTGCTAAGTCAACT<br>R: GTTGCCTCTGACATGGCTTT	JOE (G)
6	*D18S51*	X91254	213–293	18q21.33	F: GTCTCAGCTACTTGCAGG<br>R: GGAGATGTCTTACAATAACAGTTG	FAM (B)
7	*D8S315*	L12267	336-384	8q	F: CGGGAGGAAAACAAAATAGATATCG<br>R: GACCCTCAAACAAACATTGGCAA	JOE (G)
8	*SE33*	V00481	450–523	6q15	F: AATCTGGGCGACAAGAGTGA<br>R: ACATCTCCCCTACCGCTATA	FAM (B)
